# Rationale for Further Development of a Vaccine Based on the Circumsporozoite Protein of *Plasmodium vivax*

**DOI:** 10.1371/journal.pntd.0005164

**Published:** 2017-01-12

**Authors:** Anjali Yadava, Norman C. Waters

**Affiliations:** Malaria Vaccine Branch, U.S. Military Malaria Research Program, Walter Reed Army Institute of Research, Silver Spring, Maryland, United States of America; Universidade Federal de Minas Gerais, BRAZIL

## Background

*Plasmodium vivax* and its mosquito vectors have several distinctive characteristics that allow the transmission of this parasite in both temperate and tropical climates [1,2]. None, however, are as unique as its ability to form hypnozoites—dormant forms that reactivate periodically to cause multiple bouts of clinical malaria—that may be responsible for up to 80% of clinical episodes [1]. Unlike *P*. *falciparum*, which can be cured with blood schizonticidal drugs to eliminate the parasite reservoir, *P*. *vivax* needs to be treated with a combination of drugs that act on blood-stage as well as the dormant liver–stage parasites. Treatment of hypnozoites relies on a single line of 8-aminoquinoline drugs. Primaquine (PQ), the only approved drug for treating hypnozoites, causes severe hemolysis in individuals with glucose 6-phosphate dehydrogenase (G6PD) deficiency. In addition to G6PD-deficient individuals, the drug is contraindicated for the treatment of pregnant or breastfeeding women as well as infants. More recently, a deficiency in PQ metabolism that results in inadequate therapeutic levels of the active metabolite was identified in individuals that are poor or intermediate metabolizers of the drug, thus expanding the pool of subjects that cannot undergo radical cure needed for the treatment of *P*. *vivax* [3]. Thus, even if mass drug administration campaigns were a feasible option, because of the large groups of subjects that are not amenable to radical cure, vaccines will be an important component in the toolkit to prevent *P*. *vivax* malaria.

The circumsporozoite (CS) protein of Plasmodia has been the lead vaccine candidate for malaria. RTS,S, the most advanced vaccine for malaria, which is based on the CS protein of *P*. *falciparum*, underwent its first efficacy evaluation in humans adjuvanted with monophosphoryl lipid A and alum. Following promising immunogenicity (though low efficacy) in this first-in-human study [4], the vaccine was deemed suitable for further clinical testing. In 1997, Stoute and colleagues published the results of the second efficacy study conducted using a panel of more potent adjuvants [5]. A vaccine efficacy of 86% was observed using one of the adjuvants tested in this study. Almost 20 years later, in 2016, Regules and colleagues [6] published an article in which they revisited the immunization and dosing schedule used in the first study and demonstrated high levels of protection similar to those achieved in the 1997 Stoute study. In the interim, RTS,S has been tested in thousands of individuals—ranging from malaria-naïve adults in nonendemic areas to infants 6–12 weeks old in endemic areas [7]. Knowledge gained on the duration and quality of immune responses and vaccine efficacy in Phase II and III studies resulted in the design and execution of the Regules study, which led to an improvement in vaccine efficacy.

## Vaccine Efficacy Studies for *P*. *vivax*

The first-in-human efficacy study for a vaccine based on the CS protein of *P*. *vivax* was executed in 2010. As with the first efficacy study with RTS,S, the Vivax Malaria Protein 001 (VMP001) tested with GlaxoSmithKline (GSK)’s Adjuvant System AS01 resulted in high immunogenicity. In addition, it resulted in a delay in patency in 59% of vaccinees. The vaccine did not induce sterile protection [8].

## Recombinant *P*. *vivax* CSP Vaccine

VMP001 is an *Escherichia coli*–produced synthetic chimeric vaccine that incorporates all major domains of the *P*. *vivax* CS protein—namely, the amino (N-) and carboxy (C-) terminal parts of CSP and a truncated repeat region that contains repeat sequences from the immunologically divergent VK210 (type 1) and the VK247 (type 2) strains of parasites [9]. As with the *P*. *falciparum* vaccine, VMP001 underwent initial preclinical immunogenicity testing in rodents [9]. Unlike the *P*. *falciparum* vaccine, it underwent additional extensive testing and was advanced into humans following two separate, preclinical immunogenicity [10] and efficacy [11] studies conducted in nonhuman primates.

## Preclinical Efficacy Study

**Fig 1:**
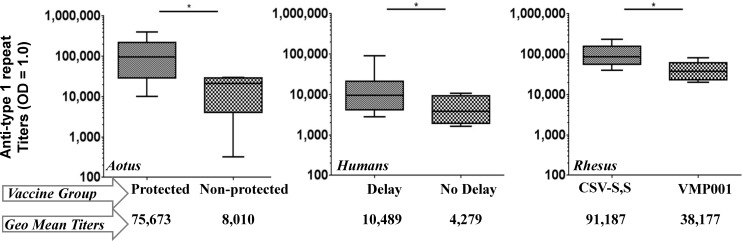
How high is high enough? In two studies assessing efficacy of the VMP001 vaccine, higher anti-type 1 repeat antibodies were associated with a positive outcome after sporozoite challenge. Protected *Aotus* (left panel; [10]) and humans with a delay to parasitemia (middle panel; [7]) had significantly higher anti-type 1 repeat antibodies. In rhesus monkeys, CSV-S,S, a particulate formulation, generated significantly higher antibodies against the type 1 repeat compared to its soluble counterpart VMP001 (right panel; [9]). These data raise the question—how high do the anti-repeat titers need to be in order to protect humans post-challenge? (Horizontal line within the box and whisker graphs represents the median values. Geometric mean titer values are listed below each group.)

*Aotus nancymaae* monkeys were used to evaluate the immunogenicity and efficacy of VMP001 formulated with a TLR9 agonist in a water-in-oil emulsion [11]. Six weeks following three immunizations, monkeys were challenged intravenously with 10,000 sporozoites from the Brazil VII strain of *P*. *vivax* (type 1), and blood stage parasitemia was monitored post-splenectomy. Vaccinated monkeys generated strong humoral immune responses. 66.7% of vaccinated monkeys demonstrated sterile protection following challenge. Protection was associated with antibodies directed against the central repeat region (Fig 1A) [11].

## Clinical Efficacy Study

Based on promising efficacy results in the nonhuman primate model, a first-in-human Phase I/IIa vaccine efficacy study of VMP001 formulated in GSK’s Adjuvant System AS01_B_ was undertaken. All volunteers generated robust immune responses to the vaccine antigen. To test vaccine efficacy, 27 vaccinated volunteers and 6 non-vaccinated infectivity controls were exposed to a *P*. *vivax* controlled human malaria infection (CHMI) two weeks after their third immunization. Vaccination did not induce sterile protection; however, a small but significant delay in time to parasitemia was seen in 59% of vaccinated subjects compared to the control group. As in the *Aotus* efficacy study, an association was identified between levels of anti-type 1 repeat antibodies and prepatent period (Fig 1B) [8].

## Preclinical Immunogenicity Study Comparing Soluble versus Particulate Formulations

To assess if the modality of antigen delivery had an outcome on immunogenicity, CSV-S,S, a particulate counterpart of VMP001, was designed in collaboration with GlaxoSmithKline. This fusion protein comprises CS protein of vivax (CSV)—which is identical to VMP001—fused to a portion of hepatitis B surface antigen and coexpressed with free S antigen in *Saccharomyces cerevisiae*. Together, these proteins assemble into virus-like particles in a manner similar to RTS,S. The immunogenicity of VMP001 and CSV-S,S, both formulated in GSK’s Adjuvant System AS01, were assessed in rhesus macaques. Both VMP001 and CSV-S,S were shown to be immunogenic in rhesus monkeys [10].

The results of two efficacy studies indicated an association between anti-type 1 repeat antibodies and protection—either sterile protection, as seen in *Aotus* (Fig 1A), or a delay to parasitemia, as seen in humans (Fig 1B). As a logical next step, anti-type 1 antibody responses were analyzed in immunized rhesus. This analysis revealed that the particulate formulation generated significantly higher anti-repeat antibody responses compared to the soluble protein (Fig 1C) [10].

The immune responses generated with VMP001/AS01 resulted in a delay to parasitemia in humans. Yet, VMP001 was able to induce sterile protection in a preclinical efficacy study. The protective efficacy of VMP001 in *Aotus* appears to have been achieved based on the induction of high antibody titers that, perhaps, were attained using a combination of potent adjuvants (Montanide plus CpG). Therefore, it indicates that the vaccine antigen may have protective capability if it were delivered with an adjuvant that was capable of inducing a higher magnitude of desired antibodies in humans. Alternatively, testing the particulate formulation CSV-S, S (which so far seems to have induced the highest anti-repeat antibody titers among all three studies) in humans may answer the question of how high is high enough. Or, further studies may inform if these titers are a red herring. The answer can only be known following additional efficacy studies. A correlation between antibody titers [12] and avidity [6] post-RTS,S vaccination was arrived at following several efficacy studies[7]. To achieve similar answers for *P*. *vivax* requires further studies that can be used to benchmark the protective correlates.

## Conclusion

Developing a vaccine for a parasite that has multiple hosts and multiples stages of life cycle within the mammalian host is not a trivial task as evidenced by more than two decades of efforts invested in RTS,S and other *P*. *falciparum* vaccines. While vaccine efforts have continued for *P*. *falciparum* over these years, *P*. *vivax*, the more complex parasite, continues to be neglected. The most recent WHO rainbow table provides consolidated information on the current status of global efforts on malaria vaccines. The table lists a total of 61 clinical studies that are considered active. Of these, 60 studies are to test various iterations of *P*. *falciparum* vaccines (30 preerythrocytic, 17 blood stage, and 13 whole sporozoite vaccines). Only one study citation pertains to *P*. *vivax* vaccine effort (http://www.who.int/immunization/research/development/Rainbow_tables/en/). While this number may be a slight underrepresentation as, in addition to our published study, Herrera and colleagues have ongoing efforts to test irradiated *P*. *vivax* sporozoite- (ASTMH 2015) and synthetic peptide–based vaccines [13], the overall dichotomy between efforts to evaluate products targeting *P*. *falciparum* versus *P*. *vivax* are starkly evident. Lessons learned from *P*. *falciparum* suggest that several efficacy studies need to be conducted to find an optimum vaccine, dosage, and schedule. The glimmer of hope seen with the first efficacy study and results from the comparative immunogenicity studies with different formulations based on *P*. *vivax* CSP indicate that further human efficacy studies are in order to generate information in support of a vaccine to prevent this form of malaria. Additional studies with vaccines that have demonstrated the ability to generate robust immune responses may pave the way for these or other vaccines that are currently under preclinical development [13–15]. While a vaccine based on a single antigen may not be sufficient, recent results on the RTS,S vaccine indicate that CS-based vaccines can confer high level of protection on their own. If not alone, such vaccines are likely to be essential components of a multi-antigen vaccine portfolio. Thus, additional testing is critical to further this important component of a vaccine platform for *P*. *vivax*. It was a controlled study that led to the identification of the role of host genetics in PQ treatment failure [3]. Additional studies may shed light on the role of different aspects of humoral or cellular immune responses as well as host genetics in infection and prevention of *P*. *vivax* malaria. It is imperative to maintain the momentum and conduct additional efficacy studies to determine if the results obtained so far are on the right track or if a course correction is needed. It is time to invest in *P*. *vivax* vaccine efforts—these investments will go a long way towards developing a vaccine for *P*. *vivax*, the still neglected though most widespread and complicated malaria parasite.
